# Role of SNAP‐25 *Mnl*I variant in impaired working memory and brain functions in attention deficit/hyperactivity disorder

**DOI:** 10.1002/brb3.2758

**Published:** 2022-09-06

**Authors:** Diangang Fang, Binrang Yang, Peng Wang, Tong Mo, Yungen Gan, Guohua Liang, Rong Huang, Hongwu Zeng

**Affiliations:** ^1^ Department of Radiology Shenzhen Children's Hospital Shenzhen China; ^2^ Development and Behavior Specialty Shenzhen Children's Hospital Shenzhen China; ^3^ Cardiac Rehabilitation Center Fuwai Hospital CAMS&PUMC Beijing China; ^4^ Department of Radiology Peking University Shenzhen hospital Shenzhen China

**Keywords:** ADHD, brain function, SNAP‐25, working memory

## Abstract

**Introduction:**

Attention deficit/hyperactivity disorder (ADHD) is a hereditary neurodevelopmental disorder characterized by working memory (WM) deficits. The *Mnl*I variant (rs3746544) of the synaptosomal‐associated protein 25 (SNAP‐25) gene is associated with ADHD. In this study, we investigated the role and underlying mechanism of SNAP‐25 *Mnl*I variant in cognitive impairment and brain functions in boys with ADHD.

**Method:**

We performed WM capacity tests using the fourth version of the Wechsler Intelligence Scale for Children (WISC‐IV) and regional homogeneity (ReHo) analysis for the resting‐state functional magnetic resonance imaging data of 56 boys with ADHD divided into two genotypic groups (TT homozygotes and G‐allele carriers). Next, Spearman's rank correlation analysis between the obtained ReHo values and the WM index (WMI) calculated for each participant.

**Results:**

Compared with G‐allele carrier group, there were higher ReHo values for the left medial prefrontal cortex (mPFC) and higher WM capacity in TT homozygote group. Contrary to TT homozygote group, the WM capacity was negatively correlated with the peak ReHo value for the left mPFC in G‐allele carrier group.

**Conclusion:**

These findings suggest that SNAP‐25 *Mnl*I variant may underlie cognitive and brain function impairments in boys with ADHD, thus suggesting its potential as a new target for ADHD treatment.

## 1 INTRODUCTION

Attention deficit/hyperactivity disorder (ADHD) is a central cognitive disorder that is common in children and has an incidence rate of approximately 7%, which is a significant increase compared to a decade ago (Christiansen et al., 2019). Children with ADHD mainly exhibit complex symptoms, including impulsiveness, inattention and hyperactivity deemed atypical for their age. These symptoms seriously impact children's behavioral performance and social relationships at school as well as those with their family. Notably, these effects often persist into adulthood. ADHD in adult has an incidence rate of up to 5% (Bonvicini et al., 2016).

Impaired working memory (WM) capacity is a key characteristic of ADHD (Fried et al., 2019). Related to this, familial and twin studies have demonstrated the influence of genetics on ADHD development (Bonvicini et al., 2016; Thapar & Cooper, 2016) and impaired WM capacity (Friedman et al., 2008), with gene variants (polymorphisms) accounting for approximately 60%−90% of cases (Liu et al., 2017). To date, candidate genes, including SNAP‐25, dopamine receptor D4 protein (DRD4), dopamine receptor D5 protein (DRD5), dopamine transporter (DAT), dopamine beta‐hydroxylase (DBH), 5‐hydroxytryptamine transporter (5‐HTT) and 5‐hydroxytryptamine (serotonin) receptor 1B (5HT1B), have been identified from quantitative and molecular genetic studies, as well as ADHD animal models (Faraone et al., 2005; Gizer et al., 2009; Russell, 2011).

Synaptosomal‐associated protein 25(SNAP‐25) is a presynaptic plasma membrane protein that is highly expressed in nerve cells (Brophy et al., 2002). SNAP‐25 has roles in synaptic vesicle docking, priming and fusion and fast calcium (Ca^2+^)‐dependent exocytosis (Catterall, 1999; Brophy et al., 2002; Mohrmann et al., 2013; Shaaban et al., 2019). Notably, SNAP‐25 has been implicated in axonal growth and synaptic plasticity, which contributes to proper cognitive function (Oyler et al., 1989; Barr et al., 2000; Martinez‐Arca et al., 2001). Other studies have reported that SNAP‐25 gene variants may alter expression levels, which can affect the fusion of synaptic vesicles and the release of neurotransmitters (Brophy et al., 2002; Hawi et al., 2013). SNAP‐25 hypo‐activity and hyper‐activity are implicated in various cognitive disorders, such as ADHD (McKee et al., 2010). However, thus far, the underlying mechanism of SNAP‐25 in these cognitive disorders has been poorly characterized. SNAP‐25 gene variants have been linked to modulate the development and plasticity of the prefrontal‐limbic network (Houenou et al., 2017). A study has shown that abnormalities in the number of synapses or structural integrity of the prefrontal cortex, possibly due to alterations in the tissue levels of the SNAP‐25 protein, may induce “hypofrontality,” which has been observed in some psychiatric disorders (Karson et al., 1999).

The human SNAP‐25 gene at the chromosome 20p11.2 locus (Maglott et al., 1996) has several variants, including rs3746544, rs363006, rs362998, rs362549, rs8636, and rs1051312 (Liu et al., 2017). In one study, reduced SNAP‐25 gene expression and spontaneous hyperkinetic behavior in the mouse coloboma (Cm) mutant strain demonstrated the importance of the SNAP‐25 gene in ADHD (Hess et al., 1992). Genetic variations that result in reduced gene expression or loss of function in mouse models suggest a potentially similar genetic etiology in humans (Barr et al., 2000). The *Mnl*I variant (rs3746544), located in the 3′‐untranslated region (3′‐UTR) of the SNAP‐25 gene, has been found to be associated with ADHD and symptom severity (Herken et al., 2014; Barr et al., 2000). However, to date, the underlying neurobiological mechanism of the SNAP‐25 rs3746544 has been poorly characterized.

A novel gene‐brain‐behavior network has been identified in which a genotype located in SNAP‐25 affects working memory and has age‐dependent effects on both brain structure and brain activity (Soderqvist et al., 2010). Resting‐state functional magnetic resonance imaging (rs‐fMRI) measures spontaneous temporal fluctuations in brain activity while the subject is at rest. Rs‐fMRI was primarily to estimate connectivity in the brain relating to a spontaneous time series. Spontaneous fluctuations in activity in different parts of the brain can be used to study brain function networks. Functional connectivity is defined as the temporal dependency of neuronal activation patterns from anatomically separated brain regions. Regional homogeneity (ReHo) describes the summarized local FC between a given node and its nearest neighboring nodes (Zang et al., 2004). Kendall's coefficient concordance (KCC) can measure the similarity of a number of time series. ReHo used KCC to measure the similarity of the time series of a given voxel to those of its nearest neighbors in a voxel‐wise way (Zang et al., 2004). The ReHo method has been used in brain functional studies of various psychiatric disorders, including ADHD, autism spectrum disorder and depressive disorder (Hao et al., 2019; Li et al., 2018; Zhou et al., 2018).

According to the previous study, boys with ADHD have WM capacities deficit, also SNAP‐25 gene affects WM. Therefore, the *Mnl*I variant (rs3746544) of the SNAP‐25 gene would also has certain affection on WM. To explore influences of *Mnl*I variant (rs3746544) on WM and its potential approach, brain network, we conduct a study, measuring intelligence, using ReHo method to analyze the rs‐fMRI data and do correlation analysis. We also speculate that the WM capacity and ReHo value may be correlated. Our findings provide novel preliminary insights into the underlying mechanisms of the SNAP‐25 *Mnl*I variant and suggest its potential as a target in ADHD treatment.

## 2 MATERIALS AND METHODS

### 2.1 Study participants

A total of 66 boys diagnosed with ADHD at the Health Department of Shenzhen Children's Hospital from July 2013 to July 2015 were enrolled in this study. On the one hand, children were eligible for enrolment if they were (1) between the ages of 8 and 10 years; (2) had an intelligence quotient of >70, as measured by the Wechsler Intelligence Scale for Children version IV (WISC‐IV); (3) of normal hearing and eyesight; (4) of Han Chinese ethnicity; and (5) right‐handed. Each study participant was assessed based on the Schedule for Affective Disorder and Schizophrenia for School‐Aged Children Present and Lifetime Version (K‐SADS‐PL) (Kaufman et al., 1997). On the other hand, children with a history of mental retardation, tic disorder, learning disabilities, conduct disorder, or any other medical conditions, including an inability to cooperate during MRI examinations or contraindications to MRI, were excluded. The study participants who were on medication, including stimulants (methylphenidate), or undergoing treatments by psychiatrists or psychologists were also excluded from the assessment. This study secured approval from the Medical Research Ethics Committee of Shenzhen Children's Hospital, China. Informed consent was obtained from all study participants and their guardians.

### 2.2 Genotyping

The SNAP‐25 rs3746544 genotyping of peripheral venous blood samples from the participants was performed using the FlexiGene DNA Kit (QIAGEN, Germany) according to the manufacturer's instructions. The following primer pairs were used: forward, *5′ TTCTCCTCCAAATGCTGTCG 3′*, and reverse, *5′ CCACCGAGGAGAGAAAATG 3′*. After DNA extraction, polymerase chain reaction (PCR) amplification was performed with EX‐Taq polymerase and GC buffer (Takara, Dalian, China). The PCR protocol started with a denaturing cycle at 94°C for 2 min, followed by 30 cycles of 94°C for 30 s, 52°C for 30 s, 72°C for 45 s and, finally, an extension step at 72°C for 8 min.

### 2.3 MRI acquisition and preprocessing

We used a 3.0T MRI scanner (Siemens Skyra) to acquire data on structural images and resting‐state fMRI (rs‐fMRI). The parameters for the three‐dimensional (3D) T1‐weighted isotropy volumetric sequence were as follows: repetition time (TR) = 2300 ms, echo time (TE) = 2.26 ms, inversion time (TI) = 900 ms, flip angle (FA) = 8°, field of view (FOV) = 256 × 200 mm^2^, acquisition matrix = 256 × 200, GRAPPA = 2, slice thickness = 1 mm without gap, slice number = 176, and acquisition time (TA) = 3 min 48 s. The settings for the collection of rs‐fMRI data based on an echo‐planar imaging sequence were as follows: TE = 30 ms, TR = 2,000 ms, phase‐encoding direction = A > > P, FA = 90°, acquisition matrix = 94 × 94, GRAPPA = 2, slice thickness = 3 mm, 32 transversal slices, 130 volumes, and TA = 4 min 28 s. Prior to the rs‐fMRI examination, participants were asked to relax, lie still, and close their eyes whilst staying awake.

### 2.4 fMRI analysis

A radiologist with 10 years’ experience of brain function study and blinded to genotypic information performed the preprocessing of the raw fMRI data using the Data Processing Assistant for Resting‐State fMRI (DPARSF 2.3; http://www.restfmri.net/forum/DPARSF), RS‐fMRI Data Analysis Toolkit (REST 1.2; http://www.restfmri.net/forum/index.php), and MATLAB‐based Statistical Parameter Mapping (SPM 8; http://www.fil.ion.ucl.ac.uk/spm). We discarded the first 10 time points of each functional time series for the resting state data corresponding to scanner calibration and magnetization equilibration. Next, the data were subjected to slice timing, head motion correction, spatial normalization to the Montreal Neurological Institute template, resampling to 3 × 3 ×3 mm^3^, temporal band‐pass filtering and regressing of nuisance signals, such as six‐head motion parameters, white matter, and cerebrospinal fluid. Another group of 10 subjects was excluded because their data had a maximum displacement of >1.5 mm in any of the cardinal directions (*x*, *y*, *z*) or a maximum spin (*x*, *y*, *z*) of >1.5°.

### 2.5 ReHo calculation

We calculated the ReHo values for each voxel of the whole brain using the REST 1.2 software (rs‐fMRI Data Analysis Toolkit; http://www.restfmri.net/forum/ index.php). The ReHo brain maps for each participant were obtained, and individual ReHo maps were generated for each data set.

### 2.6 WM capacity analysis

The WM capacity of each participant was assessed by professional technician certified by the Psychometric Professional Committee of the Chinese Psychological Association, using the WISC‐IV. The WM index (WMI) was calculated from the Digit Span and Letter‐Number Sequencing scores.

### 2.7 Correlation analysis

The medial prefrontal cortex (mPFC) of the bilateral cerebral hemispheres was defined as the region of interest (ROIs). ROIs were set based on the second level group analysis (two‐sample *t*‐test) results, defining the Peak MNI coordinate, with 3 mm radius. The ReHo peaks of each ROI in the two *Mnl*I variant genotypic groups (TT homozygote and G‐allele carrier) were then calculated. In addition, we performed Spearman's rank correlation analysis between the calculated WMI and the ReHo peak values of each ROI in the TT homozygote and G‐allele carrier groups.

### 2.8 Statistical analysis

#### 2.8.1 Demographic information analysis

Statistical analyses of the two *Mnl*I variant genotypic groups (TT homozygote and G‐allele carrier) were performed using SPSS version 18.0(SPSS Inc, IBM, USA) for Windows. The two‐sample *t*‐test was used to compare the differences between the two genotypic groups. Data were presented as the mean ± standard deviation (SD). If *p* value < .05 for the WMI between two genotypic groups, then it will be considered having statistically significant meaning.

#### 2.8.2 ReHo analysis

We used the one‐sample *t*‐test for conducting the independent assessment of each group. The two‐sample *t*‐test was used to compare differences between the two genotypic groups. Here, age was considered a covariant. A cluster of more than 10 voxels with *p* < .05 after AlphaSim correction was deemed statistically significant.

#### 2.8.3 Correlation analysis

Spearman's rank correlation analysis was performed to determine the association between the ReHo values and WMI for the two groups.

## 3 RESULTS

### 3.1 Demographic information

**FIGURE 1:**
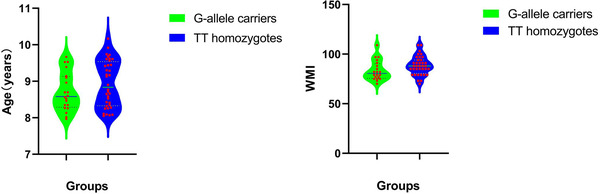
Graphs showing no significant difference in age between the two groups (left). TT homozygotes had higher WM quotients than G‐allele carriers (*t* = 2.098; *p* < .05). TT homozygotes had a higher median for the WM intelligence quotient than G‐allele carriers (right)

The 56 study participants were divided into two groups based on their SNAP‐25 *Mnl*I variant genotypes: 36 boys in the TT homozygote group and 20 boys in the G‐allele carrier group (Table 1 and Figure 1). There was no significant difference in age between the two groups (*t* = 1.396; *p* > .05). TT homozygotes had higher WM quotients than G‐allele carriers (*t* = 2.098; *p* < .05). In addition, the former had a higher median for the WM intelligence quotient than the latter.

**TABLE 1:** Demographic data of subjects in the two genotypic groups

	TT homozygotes	G‐allele carriers		
	(*n* = 36)	(*n* = 20)	*t*	*p*
Age (mean ± SD)	8.93 ± 0.63	8.71 ± 0.53	1.396	.169
WMI (mean ± SD)	88.33 ± 9.37	83.30 ± 9.90	2.098	.036*

* *p* < .05.

### 3.2 ReHo analysis

**FIGURE 2:**
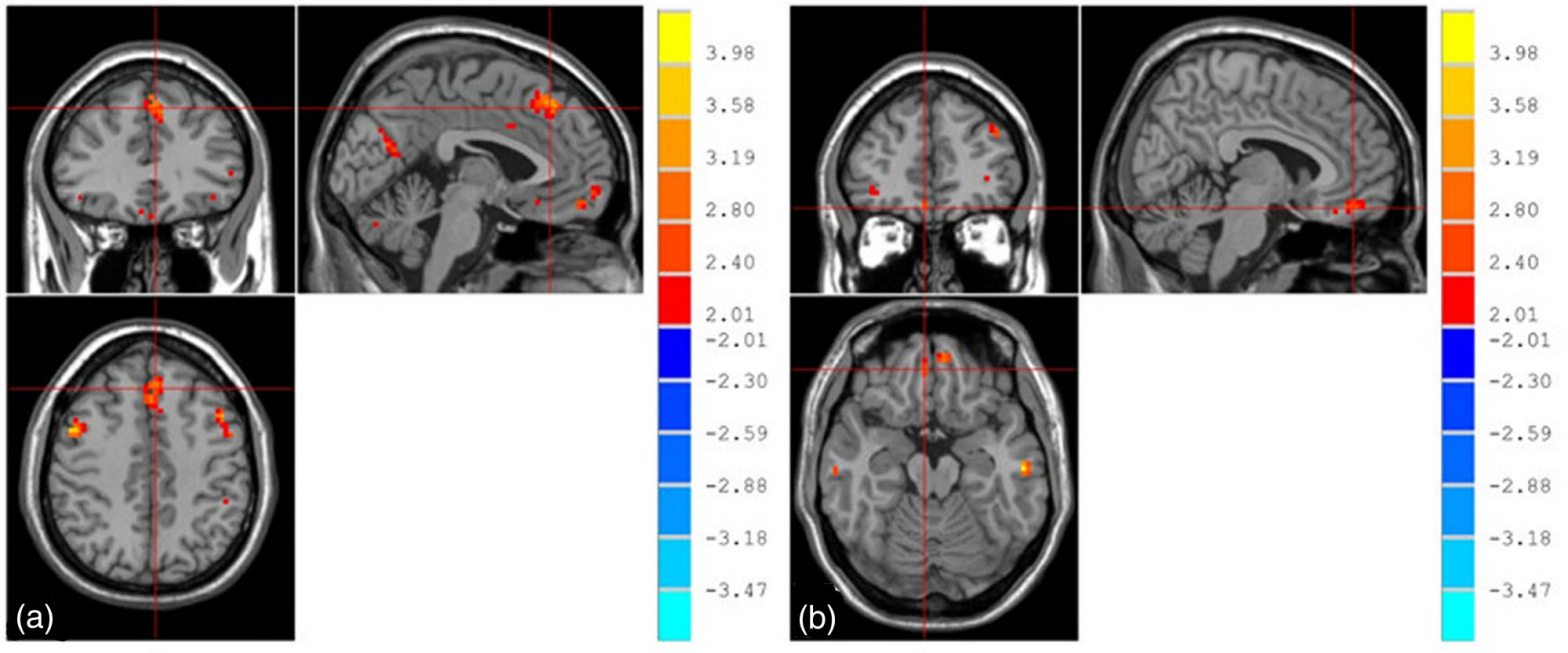
TT homozygotes showing higher ReHo values for the bilateral mPFC (a: left mPFC; b: right mPFC) than G‐allele carriers

From the two‐sample *t‐*test (AlphaSim corrected), TT homozygotes showed higher ReHo values for the bilateral mPFC than G‐allele carriers amongst boys with ADHD (*p* < .05, Table 2 and Figure 2).

**TABLE 2:** Comparison of mPFC ReHo values between TT homozygotes and G‐allele carriers

			Peak MNI coordinate			
Region	Cerebrum	Peak intensity	*X*	*Y*	*Z*	Number of voxels
mPFC	R	2.99	6	45	–18	12
mPFC	L	3.38	–3	33	45	65

*Note*: AlphaSim correction *p* < .05; cluster > 10 voxels.

### 3.3 Correlation analysis

**FIGURE 3:**
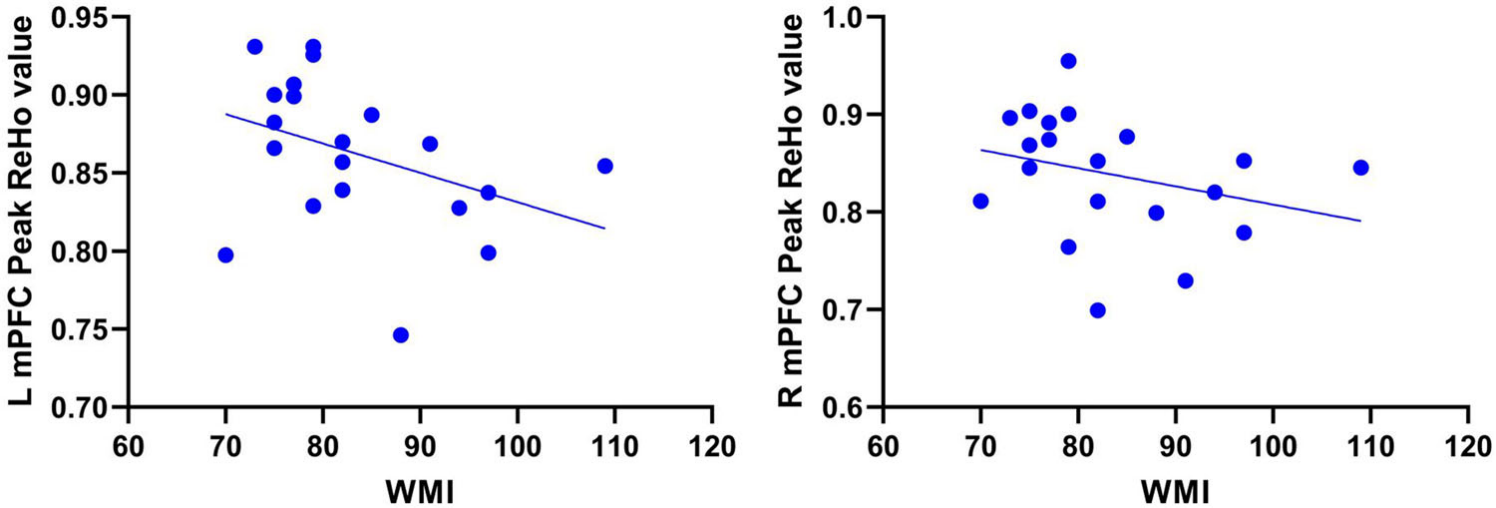
Negative correlations of individual peak values of the left mPFC with the individual WM capacities of G‐allele carriers (*r* = −0.454; *p < .05). The peak ReHo value was not correlated with cognitive function in the right mPFC of G‐allele carriers (*r* = −0.410; *p* > .05)

**FIGURE 4:**
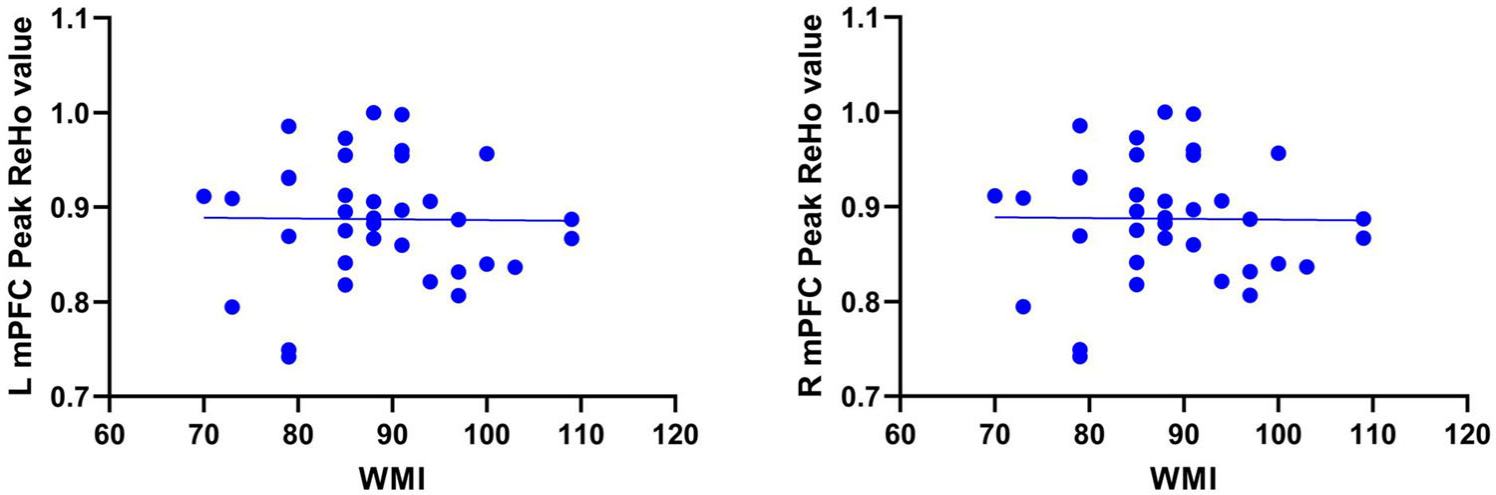
Graphs showing no correlation between peak ReHo values and cognitive function in the left mPFC of TT homozygotes (*r* = 0.062; *p* > .05). The peak ReHo value was also not correlated with cognitive function in the right mPFC of TT homozygotes (*r* = −0.097; *p* > .05)

Spearman's rank correlation analysis was adopted to determine the association between cognitive function and resting‐state brain function. The results showed that the WMI score was negatively correlated with the peak ReHo value in the left mPFC of G‐allele carriers (*r* = −0.454, *p* = .045; Table 3 and Figure 3). Moreover, the peak ReHo value was not correlated with cognitive function in the remaining ROIs (Table 3 and Figures 3 and 4).

**TABLE 3:** Results of the correlation analysis between cognitive function and resting state brain function in the TT homozygote and G‐allele carrier groups (*r*/*P*)

ROI	TT homozygote group	G‐allele carrier group
Right mPFC	0.062/0.719	−0.410/0.073
Left mPFC	−0.097/0.575	−0.454/0.045*

*r*, correlation coefficient; *P*, *p* value; *r* > 0, positive correlation; *r* < 0, negative correlation.

* *p* < .05.

## 4 DISCUSSION

In this study, ReHo analysis of the rs‐fMRI data of boys with ADHD with the SNAP‐25 *Mnl*I variant revealed that TT homozygotes had significantly higher ReHo values for the bilateral mPFC than G‐allele carriers. Moreover, lower peak ReHo values for the left mPFC can predict higher WM capacity in G‐allele carriers, but not in TT homozygotes. Thus, our findings provide novel insights into the differences between the two *Mnl*I variant genotypic groups of boys with ADHD in terms of spontaneous neuronal activities in the brain and WM capacities. Nonetheless, further studies are required to obtain deeper mechanistic insights regarding *Mnl*I variant in ADHD development.

Importantly, results showed that ReHo values for the two *Mnl*I variant genotypic groups differed in the mPFC of boys with ADHD. Previous studies revealed functional abnormalities in children with ADHD (Shanmugan et al., 2016; Wang et al., 2020). For example, Zhou et al. (2018) found that these children had significantly lower ReHo values for the right middle frontal gyrus, thereby implying fewer spontaneous neuronal activities in this region. Very interestingly, present study also showed that TT homozygotes had significantly higher ReHo values for the bilateral mPFC than G‐allele carriers. The mPFC is well characterized for its role in multiple higher cognitive functions, including decision‐making, error prediction, response selection and WM (Hauser et al., 2014; Salavert et al., 2018). The *Mnl*I variant has been found to alter the binding site of microRNAs in various cortical regions and, consequently, alter their functions in attention and inhibition (Hawi et al., 2013). Thus, we deduced that this variant may alter spontaneous neuronal activities in the mPFC.

In this study, there was higher WMI in TT homozygotes than in G‐allele carriers. Consistent with previous studies, we observed a correlation between SNAP‐25 and WM capacity, as well as a deficiency in WM (Soderqvist et al., 2010; Gao et al., 2015). The rs3746544‐induced alteration of the binding site of microRNAs may influence the SNAP‐25 expression level and lead to decreased neurotransmitter release and synaptic function, consequently impairing cognition and ultimately leading to ADHD development (Hawi et al., 2013). On the contrary, several studies have reported a higher risk for ADHD development in TT homozygotes than in G‐allele carriers (Forero et al., 2009; Ye et al., 2016; Wang et al., 2018). Furthermore, a study on autism spectrum disorders demonstrated that the lower expression levels of rs3746544 could lead to cognitive and neuropsychological deficits in children (Braida et al., 2015). Although our findings suggest a direct role for rs3746544 in ADHD pathology via SNAP‐25 gene downregulation, the underlying mechanism requires further follow‐up studies.

Notably, the other highlight point was that the WMI was negatively correlated with the peak ReHo value for the left mPFC (*r* = −0.454; *p* = .045) in G‐allele carriers, but not in TT homozygotes. As a cranial capability of temporarily storing information and controlling cognitive function, WM is often impaired in ADHD patients (Soderqvist et al., 2010; Fried et al., 2019). The mPFC has been implicated in WM development, as evidenced by increased firing frequency or synchronization in animal studies, which allows control over actions and ensures that tasks are performed effectively and efficiently (Horst & Laubach, 2012; Yang et al., 2014; Yang & Mailman, 2018). Proper regulation of the delay‐period mPFC activity is critical for information retention when performing a WM task (Liu et al., 2014).

To the best of our knowledge, we are the first group to report on the effects of SNAP‐25 *Mnl*I variant on mPFC function and WM capacity in ADHD. In particular, we found that the mPFC function was correlated with WM capacity in G‐allele carriers with ADHD. Additionally, our results support our hypothesis that SNAP‐25 *Mnl*I variant may underlie cognitive and brain functional impairments in children with ADHD. Related to this, decreased ReHo values for the left mPFC in G‐allele carriers might partly explain the poor WM capacity in children with ADHD. Additionally, lower peak ReHo values for the left mPFC predicted higher WM capacity in G‐allele carriers. Thus, our results illuminate the role of SNAP‐25 *Mnl*I variant in modulating cognitive performance and brain activity in a gene‐brain‐cognitive network. Taken together, our findings reveal the importance effect of SNAP‐25 *Mnl*I variant in mPFC through brain network, suggesting its potential as a new target in ADHD treatment.

The limitations of our study include the followings: (1) the relatively modest sample size for the MRI study due to the challenge in asking children with ADHD to listen to instructions, (2) the relatively modest sample size of the genetic study due to high cost, and (3) the narrow age range of 8−11 years, unlike other studies that included other age groups. Thus, in the future, we hope to conduct follow‐up studies with larger age groups.

Our investigation of the underlying mechanism of the SNAP‐25 *Mnl*I variant in WM capacity in ADHD children revealed insights into the role of SNAP‐25 *Mnl*I variant in the left mPFC, suggesting its potential as a novel target in ADHD treatment. Future longitudinal studies should be conducted to elucidate the changes in WM capacity and mPFC functions in response to early‐stage interventions.

## CONFLICT OF INTEREST

The authors declare no competing or conflicts of interest.

### PEER REVIEW

The peer review history for this article is available https://publons.com/publon/10.1002/brb3.2758.

## Data Availability

The data in this study are available from the corresponding author based on the reasonable request.
